# Fused Deposition Modeling of Microfluidic Chips in Polymethylmethacrylate

**DOI:** 10.3390/mi11090873

**Published:** 2020-09-19

**Authors:** Frederik Kotz, Markus Mader, Nils Dellen, Patrick Risch, Andrea Kick, Dorothea Helmer, Bastian E. Rapp

**Affiliations:** 1 Laboratory of Process Engineering, NeptunLab, Department of Microsystems Engineering (IMTEK), University of Freiburg, 79110 Freiburg, Germany; Markus.Mader@imtek.de (M.M.); Nils.Dellen@imtek.de (N.D.); Patrick.Risch@imtek.de (P.R.); Andrea.Kick@imtek.de (A.K.); Dorothea.Helmer@imtek.de (D.H.); Bastian.Rapp@imtek.de (B.E.R.); 2 Freiburg Materials Research Center (FMF), University of Freiburg, 79104 Freiburg im Breisgau, Germany; 3 FIT Freiburg Center of Interactive Materials and Bioinspired Technologies, University of Freiburg, 79110 Freiburg im Breisgau, Germany

**Keywords:** 3D printing, polymethylmethacrylate, additive manufacturing, microfluidics, fused deposition modeling

## Abstract

Polymethylmethacrylate (PMMA) is one of the most important thermoplastic materials and is a widely used material in microfluidics. However, PMMA is usually structured using industrial scale replication processes, such as hot embossing or injection molding, not compatible with rapid prototyping. In this work, we demonstrate that microfluidic chips made from PMMA can be 3D printed using fused deposition modeling (FDM). We demonstrate that using FDM microfluidic chips with a minimum channel cross-section of ~300 µm can be printed and a variety of different channel geometries and mixer structures are shown. The optical transparency of the chips is shown to be significantly enhanced by printing onto commercial PMMA substrates. The use of such commercial PMMA substrates also enables the integration of PMMA microstructures into the printed chips, by first generating a microstructure on the PMMA substrates, and subsequently printing the PMMA chip around the microstructure. We further demonstrate that protein patterns can be generated within previously printed microfluidic chips by employing a method of photobleaching. The FDM printing of microfluidic chips in PMMA allows the use of one of microfluidics’ most used industrial materials on the laboratory scale and thus significantly simplifies the transfer from results gained in the lab to an industrial product.

## 1. Introduction

Three-dimensional (3D) printing has gained great importance for rapid prototyping of microfluidic devices in the past decade, since it allows the fabrication of microfluidic chips on the laboratory scale, with the possibility to test a great variety of different chip designs early on in the development process [1,2,3,4]. Different printing technologies have been examined for 3D printing of microfluidic devices, such as stereolithography (SL), inkjet printing, multi-jet printing, two-photon polymerization, suspended liquid subtractive lithography and fused deposition modeling (FDM) [2,5,6,7,8]. On the laboratory scale these 3D printed microfluidic devices have been used in a great variety of applications: for mixers, droplet or gradient generators or active components like valves or pumps [9,10,11,12].

However, one major issue for most rapid prototyping methods in microfluidics, including 3D printing, is the conversion of a laboratory prototype to an industrial scale product [13]. 3D printing of microfluidic chips has so far been mainly realized using stereolithography printing, since it remains the method of choice for high-resolution 3D printing with affordable machinery. However, SL usually requires photocurable resins (mostly acrylic or epoxy based) which are strongly cross-linked thermosets, rendering them unsuitable for industrial replication processes like injection molding or hot embossing, which require thermoplastic materials. Due to the high surface-to-volume ratio in microfluidics, the chip material plays a major role. Therefore the transfer between lab-processes and industrial scale comes with a change in the final system behavior, which is a major issue [13]. One option to solve this problem is the use of 3D printing methods, which can process industrially relevant thermoplastic polymers already on the laboratory scale. FDM is such a 3D printing method in which a thermoplastic filament is melted, extruded through a nozzle and solidified by cooling. Furthermore, FDM is interesting since it is capable of multimaterial printing [14]. The complexity of FDM printed systems can be even extended by integration of components like membranes or electrodes by pausing the process, integrating the external components of choice before continuing the print (so called print-pause-print) [14]. Using FDM printing a variety of microfluidic concepts like mixers or chemical reactionware have already been realized [15,16]. Recently FDM printing has been used to fabricate simple channel geometries with sub-100 µm [17].

In theory, a wide range of thermoplastic materials can be processed using FDM, but only few materials have been studied. Most microfluidic chips are printed using poly (lactic acid) (PLA) or acrylonitrile-butadiene-styrene (ABS). FDM printing of cycloolefin copolymers (COC), thermoplastic urethane (PLU) or polypropylene being a notable exception [15,17]. Polymethylmethacrylate (PMMA) is one of the most important thermoplastic materials in microfluidics due to its high optical transparency and low autofluoresence combined with a high biocompatibility [18]. Furthermore, it is a rather hydrophilic material, making it interesting for capillary-driven microfluidic systems. However, PMMA is usually structured using industrial scale polymer structuring like injection molding or hot embossing [19,20]. Rapid prototyping of PMMA has been mainly conducted using subtractive processes like laser structuring or high-precision milling, as well as laboratory-scale imprinting technologies, such as solvent replication or room temperature imprinting [21,22,23,24]. PMMA prepolymers consisting of the monomer methylmethacrylate (MMA) and the polymer PMMA have been used for replication of microfluidic PMMA chips with high resolution from a variety of different master structures like polydimethlysiloxane (PDMS) or stainless steel [25,26]. Recently, we have introduced Liquid PMMA, a photocurable PMMA prepolymer which can be structured with tens of micron resolution using direct lithography [27]. However, all these rapid prototyping processes are only capable of fabricating open 2.5-dimensional microfluidic structures, which need to be closed during a subsequent bonding step. Furthermore, the integration of microstructures and biomolecular patterns is becoming increasingly important. Protein patterns with specific shapes and patterns are capable of inducing specific cellular responses and are important for the study of cellular behavior. Structured biochemical functionalization by means of photobleaching was established in the last decade and was already used for different substrate materials, like functionalized glass slides, polymer films and cellulose paper [28,29,30].

PMMA has so far not been studied as a material for FDM printing of microfluidic chips, which, however, could allow the direct printing of embedded microfluidic chip structures. In this work, we demonstrate that microfluidic PMMA chips can be directly printed using FDM with a minimum channel width of 300 µm. To ensure a high optical transparency in the region of interest, we evaluated two strategies: direct FDM printing on the print bed and printing on top of a commercial PMMA substrate. We further evaluate the influence of the nozzle distance on the transparency of the printed PMMA. Further, we show that high-resolution microstructures like microscale line-patterns can be integrated within the printed PMMA chip and that the microchannels can be easily functionalized with biomolecules. The FDM printing process allows the fabrication of a fully functional embedded microfluidic chip within 1 h, thus demonstrating that an industrially relevant thermoplastic polymer material can be structured using 3D printing. This effectively allows a direct transfer of results gained in the laboratory to an industrial scale.

## 2. Materials and Methods

### 2.1. Printing Materials

PMMA filament was purchased from materials4print, (Bad Oeynhausen, Germany). Methylmethacrylate (MMA) technical grade was purchased from VWR, (Darmstadt, Germany). Phenylbis(2,4,6-trimethylbenzoyl)phosphine oxide (BAPO), biotin (5-fluorescein) conjugate (F5B), streptavidin-Cy3 in buffered aqueous solution (STV-Cy3), phosphate buffered saline (PBS) and 2,2′-azobis(2-methylpropionitrile) (AIBN) were purchased from Sigma Aldrich, (Taufkirchen, Germany). PMMA substrates were purchased from Röhm GmbH (Darmstadt, Germany).

### 2.2. Fused Deposition Modeling

All designs were created using Autodesk Inventor Professional 2019 and exported as STL files. The STL files were imported into PrusaSlicer-2.1.0-rc for the slicing process. The FDM printer *Prusa i3 MK3S* (Prusa Research, Prag, Czech Republic) was used for printing. The printing parameters have been optimized to 3D print microfluidic channels with a minimum channel width of 300 µm. The parameters of the printing process were as follows: layer height: 50 µm (first layer: 100 µm), infill: 100%, printing speed: 30 mm/s and nozzle temperature: 230 °C. The bed temperature was increased significantly compared to other commercial filaments like PLA to 115 °C for the first layer to reduce warping effect, i.e., delamination from the print bed. During the print, the bed temperature was lowered to 110 °C for all following layers. A 45° angle was set for the infill orientation. Infill/contour overlap was set to 25% to reduce air gaps between the printed features. All parts were printed with a 0.4 mm nozzle. For high transparency of the printed microfluidic chips, the *z*-axis distance to the printing bed was reduced by doing a first layer calibration. The nozzle was moved 100 µm closer to the print bed compared to the standard printing distance. The specified standard *z*-axis distance of the nozzle is usually adjusted until the polymer sticks nicely to the print bed and is only slightly squished. Reducing the *z*-axis nozzle distance even more than specified results in broader strands, which make the FDM printed PMMA appear more homogenous and transparent (see Section 3.1). To reduce sagging during the 3D printing of embedded channels, the material extrusion was reduced by 60% while bridging the channel structures (bridge flow ratio: 0.4). By keeping the printing speed at 30 mm/s the bridging PMMA strands are stretched upon deposition which prevents excessive sagging. To evaluate different channel geometries, a series of embedded channels with different cross-section geometries were printed and compared to their original CAD geometry. The accuracy of the printable channel widths and heights were evaluated by printing open and embedded channels with varying channel widths from 0.2–1 mm and compared to the original CAD dimensions. Evaluation of the channel sizes and geometries was executed using a light microscope of type VHX 6000 (Keyence, Osaka, Japan).

To obtain microchannels with an improved transparency in the region of interest, the channels were printed directly on commercial PMMA substrates with a thickness of 2 mm. A similar strategy has been previously described for the fabrication of channels in PLA [31]. The channels were designed to be open at the bottom side, which is printed directly on the substrate. The printed PMMA structure bonds to the PMMA substrate to form sealed microfluidic channels. The *z*-axis printing height of the chip was adjusted during slicing to allow printing on the 2 mm PMMA substrate (z-offset: 2 mm). After heating and calibration, the printer was shortly paused to align and stick the substrates to the print bed.

### 2.3. PMMA Precursor Preparation

A thermally polymerized PMMA precursor was prepared by adapting a protocol described by Qu et al. [25]. The thermal initiator AIBN was dissolved in technical MMA (0.3 mg/mL) and the mixture was polymerized by heating the mixture to 93 °C within 20 min. After 15 min at this temperature the polymerization was stopped by cooling to room temperature with an ice bath. The mixture was blended with 1 wt% BAPO.

### 2.4. Casting Process

Thermally prepolymerized PMMA precursor was polymerized for 10 min between a commercial PMMA substrate and a metal microstructure using a UV-light source (Superlite 400, Lumatec, Oberhaching, Germany) at 415 nm (exposure intensity: 5.8 mW/cm^−2^). A rectangular structure (2 × 12 mm^2^) was cut out from a stripe of scotch tape (48 µm thickness), which was used as both mold and spacer between the metal microstructures and the PMMA substrate.

### 2.5. Microfluidic Experiments

In order to connect the inlets and outlet, tubing connectors were directly printed onto the microfluidic chips. Dispensing needles (1″, Vieweg, Kranzberg, Germany) were plugged into the printed connectors and fixed using epoxy glue. Colored water was pumped through the microfluidic channels using a syringe pump (Legato 210, KDScientific, Holliston, MA, USA) with a pump rate of up to 10 mL/min.

### 2.6. Contact Angle Measurements

Contact angles were measured with an OCA15 Pro (Data Physics, Filderstadt, Deutschland) using the sessile drop method. Static contact angles were measured using 5 µL water droplets at a temperature of 25 °C.

### 2.7. UV-Vis Measurements

Optical transmission of commercial PMMA and FDM printed PMMA with variable thickness was measured using a UV-visible spectrophotometer (Evolution 201, Thermo Fisher Scientific, Waltham, MA, USA).

### 2.8. Autofluorescence Measurements

Fluorescence intensity was characterized for commercial PMMA substrates and 3D printed PMMA with a thickness of 2 mm using an inverted fluorescence microscope (DMi8, Leica, Wetzlar, Germany) with Cy5, Cy3, FITC and DAPI filters and 130 ms exposure.

### 2.9. Biofunctionalization

The immobilization of proteins inside of FDM printed microfluidic channels was done similar to a previously described protocol [28]. F5B (80 µM in PBS) was injected into the channel and exposed for 3 min to 5 min at 490 nm (exposure intensity: 7.7 mW·cm^−2^). After exposure with a custom build lithography system based on a digital mirror device the channels were rinsed with distilled water, PBS and, again, distilled water [28]. The patterns were visualized by incubating the microchannel with STV-Cy3 (5 µg/mL) for 30 min. Afterwards the channels were again rinsed with distilled water and PBS. The biomolecule patterns were visualized using an inverted fluorescence microscope (DMi8, Leica, Germany). Images were analyzed using ImageJ 1.53a. Regions of interest (ROI) were selected for background and signals (triangle shapes) and were analyzed in terms of brightness. Mean values of all ROI of background and signals respectively were averaged and average values were used to calculate signal-to-noise ratio (S/N).

## 3. Results and Discussion

We optimized the printing process to fabricate microfluidic chips in PMMA. Furthermore, several microfluidic devices were fabricated and assessed. The integration of high-resolution microstructures and biofunctionalization inside the channel was demonstrated.

### 3.1. Optimum Printing Parameters and Transparency Optimization

**Figure 1:**
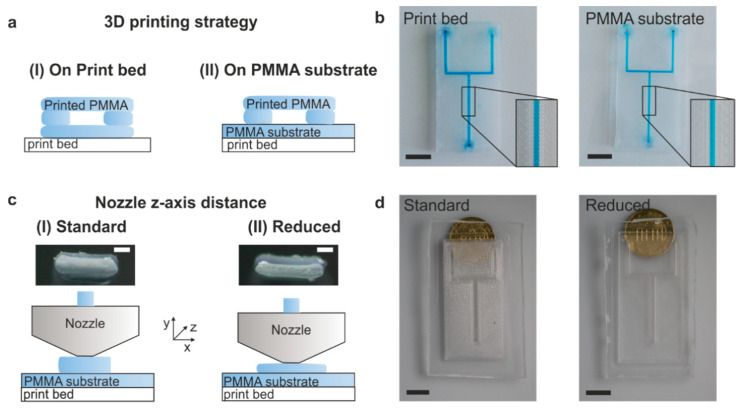
Fused deposition modeling of microfluidic chips in polymethylmethacrylate (PMMA). (**a**) Principle of printing microfluidic PMMA channels. PMMA was either directly printed on the print bed (**I**) or printed on top of a commercial PMMA slide (**II**). (**b**) Comparison of two identical microfluidic chips directly printed on the print bed and printed on a commercial PMMA substrate, respectively. As can be seen, the transparency in the region of interest is increased by printing on a commercial PMMA substrate (scale bars: 10 mm). (**c**) Increasing the optical transparency by reducing the nozzle distance: (**I**) standard configuration, (**II**) printing with a reduced nozzle distance which flattens the extruded filament. The nozzle is moving in the z-direction. The images show the 3D printed cross-section of the first layer calibration (scale bar: 100 µm). (**d**) Comparison of microfluidic channels printed with standard configuration and with reduced nozzle distance. As can be seen the transparency is increased for the printing setup with reduced nozzle distance (scale bar: 10 mm).

To print PMMA microfluidic chips with an optical transparency in the region of interest, we studied two different printing strategies: (1) standard printing procedure with direct printing on the print bed and (2) printing onto a commercial PMMA substrate. Both strategies and the respective printed microfluidic channels are shown in Figure 1a,b. First, we printed PMMA microfluidic chips using the standard printing setup, where the PMMA is deposited directly on the print bed. To ensure a completely sealed microfluidic structure, the printed channels are embedded in the PMMA chip, which is printed using 100% infill for high transparency. A minimum of two layers (0.15 mm) were deposited before printing the actual channel structures. This allows printing of embedded, leak-proof and transparent microfluidic chips, as shown in Figure 1b. However, the deposited PMMA strands from the two bottom layers are clearly visible and therefore reduce the transparency of the printed microfluidic chips. In the second strategy, we printed an open channel structure directly onto a commercial, highly transparent PMMA substrate. The heated PMMA structure bonds to the commercial PMMA substrate upon deposition and therefore allows printing of fully functional, embedded and transparent microfluidic channels. The absence of FDM printed PMMA layers between the printed channel structures and the commercial PMMA substrate allows 3D printing of PMMA microfluidics with a higher transparency than with the standard printing procedure.

We further optimized the printing process to improve the overall transparency of the FDM printed PMMA. It was found that the *z*-axis distance of the nozzle for printing the first layer has a significant impact on the observed transparency of the FDM printed PMMA. Printing with the standard parameters for *z*-axis distance, specified by the Prusa manual, PMMA with poor optical transparency is obtained. Reducing the first layer *z*-axis distance of the nozzle by 100 µm compared to the standard parameters (Prusa manual) results in PMMA microfluidic devices with significantly higher transparency (see Figure 1c,d). The reduced nozzle distance results in slightly broader deposited PMMA strands that fuse together without having gaps in between thus increasing the transparency of the FDM printed component significantly.

**Figure 2:**
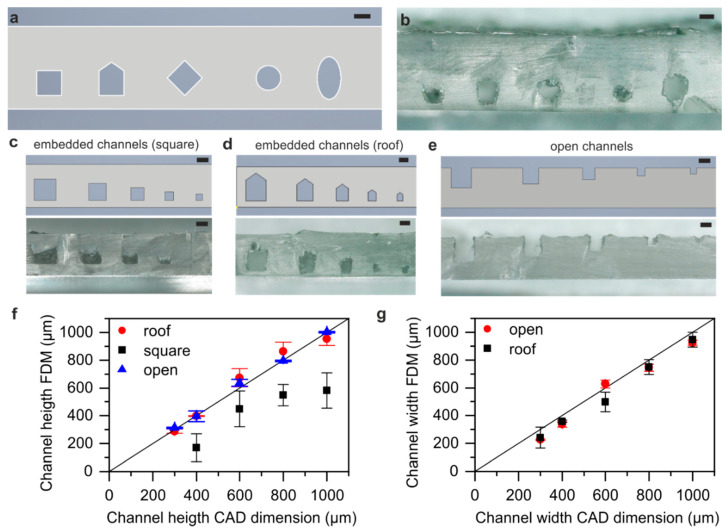
Characterization of the channel cross-section of a printed PMMA chip. (**a**) Image of the design of the printed channel cross-sections. (**b**) Different channel cross-sections with a channel width/diameter of 600 µm printed in PMMA. (**c**) Fused deposition modeling (FDM) printed embedded channels with a square cross-section for analysis of the printed channel height. Due to sagging the channel height deviates strongly from the original CAD design. (**d**) FDM printed embedded channels with a roof shaped cross-section for analysis of printed channel height and width. The addition of the roof compensates the sagging resulting in square channels. (**e**) Open square cross-section channels for analysis of the channel height and width. (**f**) Comparison of channel heights of embedded FDM printed channels using square and roof shaped cross-sections shown in (**c**,**d**) and open channels shown in (**e**) with the designed CAD heights. (**g**) Channel width of open and embedded roof shaped FDM printed channels from (**d**,**e**) compared to the designed CAD widths. (Scale bars (**a**–**e**): 500 µm).

We printed different channel cross-sections to evaluate the printing accuracy of these cross-sections and to determine the best cross-section for printing more complex embedded microfluidic channels. The CAD design and its respective printed PMMA chip can be seen in Figure 2a,b. FDM printing of a square cross-sections results in an excessive sagging of the top layer, which will clog the channel if small channel sizes are printed. The same effect was found for circular cross-sections. A major improvement was found when adding a triangular shaped roof on top of the square cross-section. The roof hereby compensates for the sagging effect and results in the square channel cross-sections. A similar optimization was found for elliptical and diamond shaped channels. Since the additional roof resulted in the most accurate microfluidic channels, all further structures were printed using this design.

The accuracy of the printed channel dimensions was evaluated by printing open and embedded (roof and square cross-section) test channels of 1 mm, 800 µm, 600 µm, 400 µm and 300 µm channel width and height, respectively (Figure 2c–e). The measured height of the resulting channel cross-sections compared to the CAD dimensions are shown in Figure 2f. Printing embedded square cross-sections resulted in excessive sagging which reduced the accuracy of the printed height compared to the CAD design significantly. Adding a roof on top of the square cross-section compensated for the sagging and allowed 3D printing of square channels with their channel heights corresponding to the square CAD design without the added roof. The open channels showed nearly no deviation from the CAD design. Both the open and embedded roof cross-section showed a high accuracy down to a channel height of 300 µm.

We further compared the width of the resulting channel cross-sections to the CAD dimensions (see Figure 2g). The actual printed channel widths of the open and roof-shaped cross-section channels are only slightly smaller than their respective CAD dimensions. This small deviation can be explained by the employed printing setup. Due to the reduced nozzle distance, used for printing of high transparency structures, the deposited PMMA strands are pressed to a slightly broader size than estimated by the slicing software. Printing of channels with a width and height below 300 µm resulted in partial clogging and was therefore not further investigated.

### 3.2. 3D Printed Microfluidic Devices

**Figure 3:**
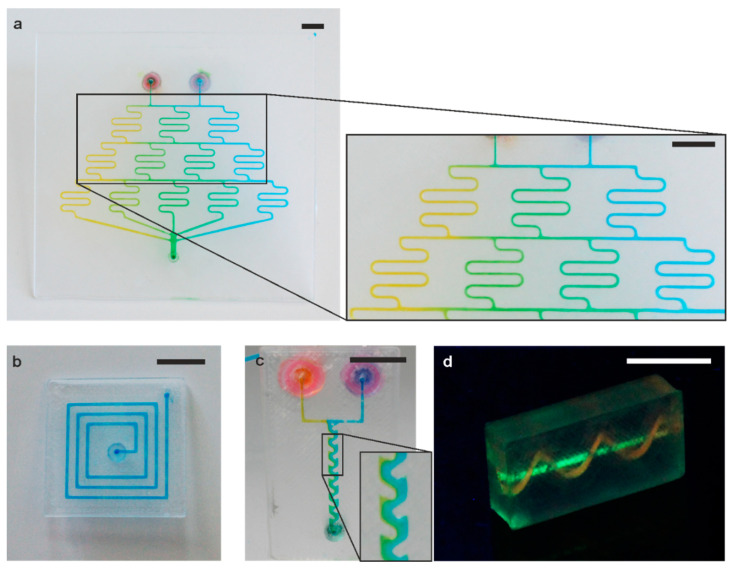
Microfluidic chips printed in PMMA. (**a**) Microfluidic mixer cascade for effective mixing of dyed water (scale bar: 10 mm). (**b**) 2.5-dimensional microfluidic spiral (scale bar: 10 mm). (**c**) Enhanced mixer structure of 600 µm periodic structures, effectively mixing dyed water (scale bar: 10 mm). (**d**) Three-dimensional microchannel printed in PMMA around a straight channel, visualized using an aqueous fluorescent dye (scale bar: 10 mm).

We printed several exemplary microfluidic devices to demonstrate the versatility of FDM 3D printing of PMMA (see Figure 3). An exemplary serpentine microfluidic mixer with a channel diameter of 600 µm was printed. It shows effective mixing along the channel cascade, resulting in a color gradient from yellow to blue (see Figure 3a). We further printed a simple microfluidic spiral with a channel width of 600 µm using the roof-shaped channel cross-section CAD design (see Figure 3b). We also show an enhanced mixer which consists of a 2 mm wide channel with 600 µm periodic geometries reaching into the channel (see Figure 3c). All three designs were printed using the roof-shaped channel cross-section CAD design. The mixers in Figure 3a,c show effective mixing of blue and yellow colored water. To demonstrate the feasibility of truly three-dimensional geometries, we also printed a 3D microfluidic spiral with 1.2 mm cross-section, which is intertwining around a straight channel (see Figure 3d). To test their performance, the printed channels were flushed with liquid at a flow rate of up to 10 mL/min for several minutes. No leakage occurred during these experiments and none of the channels showed clogging.

### 3.3. Integration of High-Resolution Microstructures

**Figure 4:**
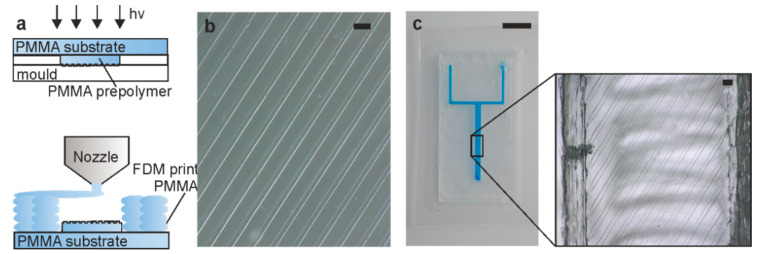
Integration of high-resolution PMMA structures inside a FDM-printed channel structure. (**a**) Principle of integration of high-resolution microstructures inside the FDM printed channel. The microstructure is fabricated by polymerizing a PMMA prepolymer directly on a commercial PMMA substrate using a lines-and-space structure. The PMMA substrate is then aligned and fixed within the FDM printer and the microfluidic channel is printed around the microstructure. (**b**) Polymerized PMMA microstructure fabricated using photocurable PMMA prepolymers (scale bar: 100 µm). (**c**) FDM printed PMMA chip including the microstructure from (**b**) (scale bar: 10 mm). The inset shows the integrated microstructure (scale bar: 100 µm).

For a broader application scope, we investigated the potential to incorporate microstructures within 3D printed channels. In order to do so, a process was developed to allow for the integration of microstructures into the FDM printed microfluidic structures (see Figure 4a). To generate the desired microstructures, we used a photocurable PMMA prepolymer, which was structured on top of a commercial PMMA substrate. This PMMA substrate was then used as the PMMA substrate plate for FDM printing. This requires careful alignment of the PMMA substrate with the PMMA microstructure in the FDM printer to allow a precise print-on process. We first replicated a microscale lines-and-space structure using the PMMA prepolymer (Figure 4b), and then printed a channel around it, resulting in a channel with integrated microstructures (see Figure 4c). By doing so, we achieved the integration of high-resolution PMMA microstructures within the FDM printed PMMA channels.

### 3.4. Characterization of Printed PMMA

The wetting behavior of the chip material is important in microfluidics, to enable, e.g., filling of the channels and thus capillary-driven microfluidics in general. Therefore, the contact angle of water was measured on a commercial PMMA substrate and on a 3D printed substrate to characterize the wetting behavior. The printed PMMA and the commercial PMMA showed a water contact angle of 69°, respectively. These values are in good accordance with literature references which have been reported to be between 65° and 72° [32] and underline the hydrophilic character of the material.

**Figure 5:**
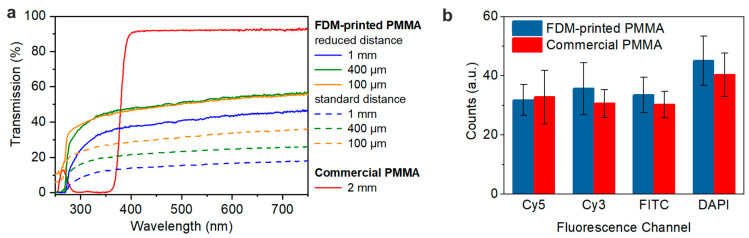
Characterization of FDM printed PMMA. (**a**) UV-Vis measurement of printed PMMA using different printing strategies (standard and reduced nozzle distance) and commercial PMMA substrates. (**b**) Fluorescence intensity of commercial PMMA substrate and FDM printed PMMA.

The optical transmission of the 3D printed PMMA with the different nozzle distances described in Section 3.1, was characterized using UV/Vis measurements (see Figure 5a). Samples with a thickness of 100 µm, 400 µm and 1 mm were measured. The transparency was compared to the optical transmission of commercial PMMA with a thickness of 2 mm, which was used as the substrate for 3D printing of microfluidic channels directly on top of the substrate. The optical transparency of FDM printed PMMA parts was increased by using a printing strategy with reduced nozzle distance compared to the standard distance. The optical transmission, however, is still below that of commercial PMMA substrates. This is why for applications where high optical transmission is required in the region of interest, the demonstrated strategy of printing on top of a commercial PMMA substrate is preferred.

To ensure the applicability of 3D printed PMMA for fluorescence-based assays, the influence of autofluorescence of the material was investigated using an inverted fluorescence microscope. The printed and the commercial substrate show comparable low autofluorescence values (see Figure 5b).

### 3.5. Biofunctionalization Inside the Printed Microfluidic PMMA Chips

**Figure 6:**
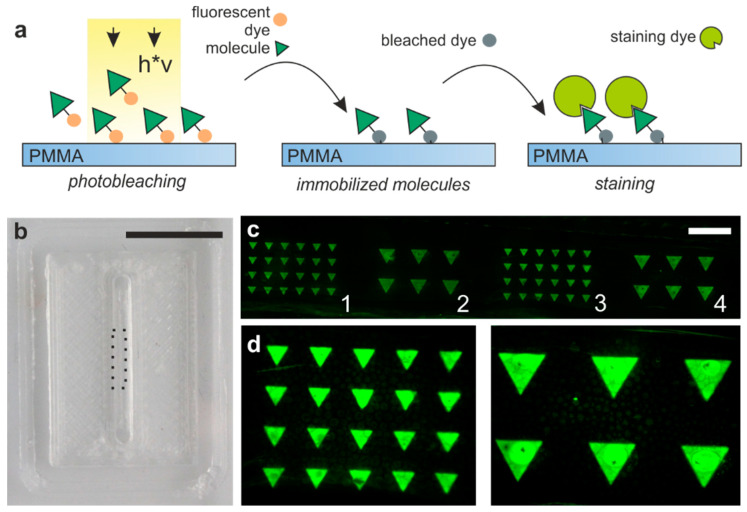
Biofunctionalization inside the 3D printed PMMA channel. (**a**) Fluorescently labelled biomolecules can be covalently bound to surfaces by photobleaching the fluorophore upon exposure with light patterns. (**b**) 2 mm wide embedded PMMA channel printed on a PMMA substrate with biofunctionalization inside the printed channel indicated by dotted lines (scale bar: 10 mm). (**c**) The bottom surface of the 3D printed channel on top of the commercial PMMA slide was functionalized with different biotin patterns by photobleaching of F5B. Patterns were illuminated for 3 min (patterns 1 and 2) and 5 min (patterns 3 and 4). The patterns were visualized via staining with STV-Cy3 (scale bar: 750 µm). Signal-to-noise ratios were calculated for all four images and were on average 29 for 3 min illumination and 11 for 5 min. (**d**) Close-up of patterns which were illuminated for 3 min.

Protein patterns with specific shapes and patterns are capable of inducing specific cellular responses and are important for the study of cellular behavior. To demonstrate the possibility to locally create these patterns within the printed PMMA channels, we used a fast protein patterning method based on the immobilization of fluorescently labeled molecules via photobleaching which we have previously reported [28]. The photochemical immobilization of fluorescently labelled biomolecules is based on the transformation of fluorophores into reactive radicals as a consequence of photobleaching upon irradiation at the absorption wavelength. The radical species, which are only formed in the exposed areas, react with functional groups and thus bond covalently to the surface. The method is shown in Figure 6a. In this work, we created the pattern directly within the channel by printing a microfluidic PMMA channel on top of a transparent PMMA substrate (shown in Figure 6b), filling it with fluorescently labelled biotin (F5B) and illuminating it with a maskless projection lithography system. The patterns of photobleached F5B were visualized by staining with STV-Cy3. Biomolecule patterns with feature sizes of 125 µm were fabricated inside the microfluidic channel showing the facile biofunctionalization of 3D printed PMMA microfluidics (Figure 6c,d).

## 4. Conclusions

In this work, we demonstrated that microfluidic PMMA chips can be fabricated down to channel diameters of 300 µm using a benchtop FDM machine. It was shown that the transparency of the printed chips can be significantly improved by using a commercial PMMA substrate as the bottom substrate for the printing process. The 3D printed PMMA was compared to commercial PMMA and shows the same low autofluorescence and hydrophilic wetting behavior, which is important for biocompatibility and channel wetting. We demonstrated that the integration of microstructures, as well as biofunctionalization in 3D printed PMMA channels, is possible. We showed that high-resolution microstructures can be produced using a simple prepolymer-casting process and that these structures can be easily integrated into the channel by directly printing onto the structured PMMA substrate. We have also shown that patterns of biomolecules can be attached to the commercial PMMA substrate using a photobleaching process. This effectively enables the formation of microfluidic channels with high-resolution biofunctionalization within the channel itself.

This work will allow the use of PMMA, one of the most important and industrially-relevant thermoplastic polymer materials in microfluidic mass-market manufacturing, in early stages of component development, where rapid prototyping and 3D printing facilitate fast design iterations and rapid experimental verifications. This closes an important gap in the academia-to-industry transition by allowing usage of the same material both during early-stage lab-scale development and industrial-scale mass-market production.
